# New insight for early detection of Alzheimer's disease

**DOI:** 10.1002/alz.083679

**Published:** 2025-01-09

**Authors:** Sharmistha Dey

**Affiliations:** ^1^ All India Institute of Medical Sciences, New Delhi India

## Abstract

**Background:**

Recent research on Alzheimer’s disease (AD) has highlighted that the oxidative damage is the earliest event of disease. These oxidative modifications are closely associated with inflammatory molecules. It is necessary to explore these two pathways with AD pathophysiology and targeted for therapeutic intervention. At present, the most validated AD biomarker is Aβ levels in the cerebrospinal fluid (CSF) and tau PET scan. None of the techniques can be used to monitor the disease with the response of treatment. The present study focussed on novel molecules in these two pathways for establishing blood‐based biomarker and therapeutic interventions.

**Method:**

Blood samples were collected from 46 AD patients, 37 mild cognitive impairment (MCI) patients and 35 geriatric‐control (GC). The levels of inflammatory molecules (LOX‐5, LOX‐12, GSK‐3β, p53, NFkb) and antioxidant molecules (FOXO, Sestrin, Sirtuins) were measured by using surface plasmon resonance and verified using Western blot in serum from AD, MCI, and GC. Statistical analysis, including Receiver Operating Characteristic (ROC), was done for further affirmation with all demographic data and clinical assessment score. The Alzheimer’s disease model neurotoxic SH‐SY5Y cell line was treated with antioxidant plant extract for rescue effect on SH‐SY5Y to see the effect on expression level of above mentioned proteins.

**Result:**

The significant alteration of some of these proteins were observed in the blood of AD patients compare to MCI and geriatric control. These proteins also showed positive correlation with the known hallmark Tau, pTau and ab‐amyloid in the study group. A significant (p<0.0001) downhill correlation was found between Tau as well as p‐Tau181 levels with HMSE and MoCA score. The treatment with antioxidant plant extract showed rescue effect of neurotoxicity by enhancing antioxidant enzymes thereby decreased reactive oxygen species (ROS).

**Conclusion:**

These proteins can serve as potential blood markers for the diagnosis of AD and supplementary antioxidant molecules can regulate and suppress their level by rescuing the neurotoxicity. This work has translational value and clinical utility in the future.

